# SNP Diversity in *CD14* Gene Promoter Suggests Adaptation Footprints in Trypanosome Tolerant N’Dama (*Bos taurus*) but not in Susceptible White Fulani (*Bos indicus*) Cattle

**DOI:** 10.3390/genes11010112

**Published:** 2020-01-19

**Authors:** Olanrewaju B. Morenikeji, Anna L. Capria, Olusola Ojurongbe, Bolaji N. Thomas

**Affiliations:** 1 Department of Biomedical Sciences, Rochester Institute of Technology, Rochester, NY 14623, USA; obmhst@rit.edu (O.B.M.); alc8941@rit.edu (A.L.C.); 2 Department of Medical Microbiology and Parasitology, Ladoke Akintola University of Technology, Osogbo P.M.B 4000, Nigeria

**Keywords:** cattle, immune response, *CD14*, single nucleotide polymorphisms, N’Dama, White Fulani

## Abstract

Immune response to infections has been shown to be mediated by genetic diversity in pattern recognition receptors, leading to disease tolerance or susceptibility. We elucidated naturally occurring variations within the bovine *CD14* gene promoter in trypanosome-tolerant (N’Dama) and susceptible (White Fulani) cattle, with genomic and computational approaches. Blood samples were collected from White Fulani and N’Dama cattle, genomic DNA extracted and the entire promoter region of the *CD14* gene amplified by PCR. We sequenced this region and performed in silico computation to identify SNP variants, transcription factor binding sites, as well as micro RNAs in the region. *CD14* promoter sequences were compared with the reference bovine genome from the Ensembl database to identify various SNPs. Furthermore, we validated three selected N’Dama specific SNPs using custom Taqman SNP genotyping assay for genetic diversity. In all, we identified a total of 54 and 41 SNPs at the *CD14* promoter for N’Dama and White Fulani respectively, including 13 unique SNPs present in N’Dama only. The significantly higher SNP density at the *CD14* gene promoter region in N’Dama may be responsible for disease tolerance, possibly an evolutionary adaptation. Our genotype analysis of the three loci selected for validation show that mutant alleles (*A/A, C/C,* and *A/A*) were adaptation profiles within disease tolerant N’Dama. A similar observation was made for our haplotype analysis revealing that haplotypes H1 (*ACA*) and H2 (*ACG*) were significant combinations within the population. The SNP effect prediction revealed 101 and 89 new transcription factor binding sites in N’Dama and White Fulani, respectively. We conclude that disease tolerant N’Dama possessing higher SNP density at the *CD14* gene promoter and the preponderance of mutant alleles potentially confirms the significance of this promoter in immune response, which is lacking in susceptible White Fulani. We, therefore, recommend further in vitro and in vivo study of this observation in infected animals, as the next step for understanding genetic diversity relating to varying disease phenotypes in both breeds.

## 1. Introduction

In recent years, the increasing availability of extensive sequencing data for livestock species due to the reduction in genome sequencing cost has provided an opportunity for comprehensive elucidation of genetic diversity and selection sweeps among and between species [1,2]. Identification of single nucleotide polymorphisms (SNP) has been a beneficial strategy to unravel selection footprints within genomic regions that are potentially associated with phenotypic variation due to evolutionary adaptation [3]. Cattle breeds with differing genetic diversities have evolved due to different environmental pressures, including heat stress, infectious diseases, and harsh climate, to the end, developing unique gene variants or footprints of adaptation allowing them to cope with environmental or disease challenges [4,5,6]. It is now known that host response to pathogenic insults can result in adaptation or counter-adaptation, requiring the immune system to respond to intruding pathogens [5,7]. Some studies have shown that bovine disease tolerance comprises a better capacity to control parasitemia and limit anemia development [8,9], which we postulate to be the opposite in susceptible cattle breeds. 

A proper innate immune response is imperative to overcome a pathogenic challenge or confer disease tolerance. Therefore, variants of the genes mediating innate immune pathway may play a crucial role in tolerance or susceptibility to disease. The cluster differentiation antigen 14 (*CD14*) gene serves as a pattern recognition receptor for lipopolysaccharide (LPS) and acts in concert with Toll-like receptors (TLRs) to facilitate detection of bacterial molecules [10]. *CD14* is a 55kD glycosyl phosphatidylinositol-anchored surface protein majorly expressed on monocytes, polymorphonuclear leucocytes, and macrophages [11,12]. Published reports have revealed the importance of *CD14* polymorphisms as candidates for studying diseases such as bovine tuberculosis and mastitis [13,14,15]. Studies have localized bovine health-related quantitative trait loci (QTL) to the genomic region of the *CD14* loci [13,15]. In addition, results from malaria studies have shown *CD14* promoter polymorphism to mediate an adaptive protective mechanism against severe disease [16,17], including regulation of parasitemia [18]. 

Cattle provide an important source of income to small-holder farmers in sub-Saharan Africa, however infectious diseases pose a major challenge to the industry through economic losses and concerns on human health [9,19]. Certain indigenous cattle breeds have acquired tolerance to a number of diseases through a better capacity to control parasitemia and limit anemia development [4,8]. An in-depth understanding of the genetic diversity inherent responsible for providing tolerance or susceptibility to indigenous breeds will provide an opportunity to elucidate mechanisms of resistance to trypanosomosis and develop marker-assistant selection strategies to develop more tolerant cattle. For example, N’Dama cattle (taurine) are naturally tolerant to the pathogenic consequences of trypanosomosis, by a better capacity to control parasitemia and limit anemia development, while White Fulani cattle (indicine) on the other hand, are comparatively more susceptible to disease development [4,6]. To initiate a proper innate immune response to infection, pattern recognition receptors such as *CD14*, and signaling molecules such as Toll-like receptors (TLR’s), are of significant importance, with the former presenting either as mCD14, expressed primarily on macrophages, dendritic cells and neutrophils, or sCD14 expressed in serum [20]. The expression regulation of *CD14* gene is therefore critical for the recognition of bacterial lipopolysaccharide during innate immunity [13,15], particularly since *CD14* mutant variant (-159T/T) expresses significantly higher soluble *CD14* levels in serum than the homozygous or heterozygous genotypes [21]. We have utilized qualitative data on *CD14* gene promoter to decipher immune response and susceptibility to malaria infection [18], showing a defective response by mutant variants, driving a cascade that worsens disease and multiple deleterious outcomes [22,23,24]. In addition, we have also shown that there are evolutionarily conserved microRNAs involved in the *CD14*-mediated immune response during bovine trypanosomosis [25], demonstrating their significance in regulating gene expression [26]. Hence, there is a need to identify and characterize naturally occurring variations within the bovine *CD14* promoter of cattle breeds that may be associated with disease tolerance or susceptibility. To this end, we elucidated the signatures of adaptation within the *CD14* gene promoter of trypanotolerant N’Dama and trypanosusceptible White Fulani cattle breeds. 

## 2. Materials and Methods

### 2.1. Animal Sampling

We sampled at random a herd of apparently healthy animals (*n =* 40; ~4–5 years old, raised in the same location, fed the same food and similar environmental exposure), comprising of 25 trypanosusceptible White Fulani and 15 trypanotolerant N’Dama cattle from south-western Nigeria. Blood samples collected from these animals for routine monitoring, Teaching and Research farm, Federal University of Technology and Federal College of Agriculture, Akure were utilized for our study. 

### 2.2. Genomic DNA Extraction, PCR, and DNA Sequencing

We extracted genomic DNA from 200µL of whole blood utilizing the Isolate II Genomic DNA extraction kit (Bioline USA Inc., Swedesboro, NJ, USA), following the manufacturer’s instructions; a final volume of 100 µL eluted DNA was stored at 4 °C until use and concentration was quantified with a spectrometer (PG Instruments Ltd, England, UK). Specific primer pairs were designed with Primer Express (version 4.0) to amplify the ~1.6 kB promoter region of bovine *CD14* gene (Supplementary Table S1). Using primer pairs and EconoTaq Plus Green 2X PCR master mix (Lucigen Corporation, Middleton, WI, USA), we amplified 1 μL of genomic DNA, optimizing reactions to a final volume of 25 μL [16]. The PCR conditions were programmed as follows: 94 °C for 2 min, and 35 cycles of 94 °C for 30 s, 59 °C for 30 s, 72 °C for 50 s, then the final extension for 5 min at 72 °C. Five microliters of amplified products were examined and band size was determined with a GeneRuler 100bp Plus DNA ladder (Supplementary Table S1). Amplified PCR products (*n =* 40) were purified with QIAquick PCR purification kit (Qiagen Inc., Valencia, CA, USA), and 2 μL of purified products were prepared for Sanger sequencing (Genewiz, South Plainfield, NJ, USA).

### 2.3. Sequence Processing and Alignment

Gene sequences from individual animals were processed and assembled with Lasergene program, version 4.0 (DNAStar, Madison, WI, USA). Sequence contigs were submitted to the GenBank and assigned accession numbers: MK358466 for N’Dama and MK358467 for White Fulani. A BLAST search of contigs from both breeds was carried out using Ensembl BLAST/BLAT genomic sequence tool (http://www.ensembl.org/Tools/Blast/GenomicSeq) to locate the genomic position of our sequences on the bovine genome ARS-UCD1.2 assembly of the Ensembl database. Sequence alignment was performed to identify regions of similarity, indicative of functional, structural, or evolutionary relationships, between *CD14* gene promoter regions of both breeds with the Ensembl program alignment tool [27].

### 2.4. Variant Analysis, Prediction of Transcription Factor Binding Sites, and miRNAs

We identified common and unique variants between N’Dama and White Fulani contigs, as well as putative single nucleotide polymorphisms through alignment (Table 1) and visual inspection using variant finders in the Ensembl browser and the Genomatix variance analysis software, version 3.10 (Genomatix, Munich, Germany). To identify functional elements such as transcription factor binding sites (TFBSs) and micro RNAs (miRNAs), we predict the effect of each identified single nucleotide polymorphism and TFBS from the sequences with the variant analysis and MatInspector program (http://www.genomatix.de/matinspector). With the *CD14* gene promoter region as target, we predict the possible miRNAs binding sites within the core promoter region with miRWalk [28,29]. We confirmed the identified miRNAs with two other prediction algorithms and databases; miRBase [30] and TargetScan [31]. We selected candidate miRNAs from these databases to analyze further, as previously described [25,26].

### 2.5. Functional Analysis of miRNAs at the Core Promoter of CD14 Gene 

In order to identify the functions and examine the possible variations in the biological process regulated by *CD14* promoter miRNAs between the two breeds, we identified miRNAs and their functions using mirPath v.3. This is a web server that provides accurate prediction for both experimentally validated miRNA and target gene interactions [32,33]. Furthermore, we searched the gene ontology database with each miRNA to ascertain relevant biological processes. 

### 2.6. Validation of Selected CD14 Gene Polymorphism by Taqman SNP Genotyping Assay

In further analysis, we randomly selected three specific SNPs (rs721906237 [C/A], rs723566082 [G/C], and rs799300279 [A/G]) from N’Dama’s *CD14* promoter sequences and custom Taqman SNP genotyping assays were designed (Thermo Fisher Scientific, Waltham MA). A total of 103 DNA samples from unrelated N’Dama animals were genotyped using the Taqman assays. Briefly, 10 µL total reaction mixture, prepared as instructed, were amplified on a CFXconnect real time PCR machine (Bio-Rad, Hercules, CA, USA), with the reaction condition as: 90 °C for 10 min, 90 °C for another 30 s, annealing at 56 °C for 30 s, extension at 72 °C for 50 s, and the final extension at 72 °C for 5 min. The CFX Manager software was used to call the allele genotypes in the samples.

### 2.7. Statistics

We performed allelic and genotypic frequency estimates from the Taqman SNP genotyping assay results using SNPStats, and tested for deviation from Hardy–Weinberg equilibrium, as described [34]. In addition, we estimated haplotype frequencies among the three loci in N’Dama *CD14* promoter. 

## 3. Results

### 3.1. Sequence Quality Control and Alignments

**Figure 1:**
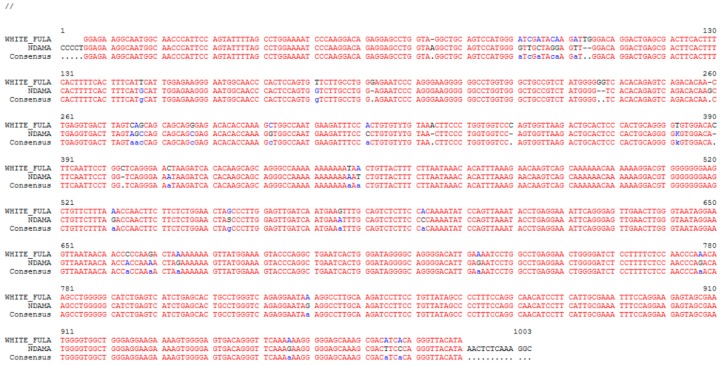
Alignment of the *CD14* gene promoter nucleotide sequences of N’Dama and White Fulani. Blue letters within alignment indicate sites of sequence diversity between animal breeds.

*CD14* promoter raw sequences trimmed to remove low quality sequence calls were removed. Forward and reverse sequences from N’Dama and White Fulani were individually processed, assembled, and contigs were made with SeqMan Pro of the Lasergene program, version 4.0 (DNAStar, Madison WI, USA). The contigs from both breeds were individually used to search the bovine genome ARS-UCD1.2 assembly [26] (Figure 1).

### 3.2. Nucleotide Mapping, SNP Identification, and Classification

**Figure 2:**
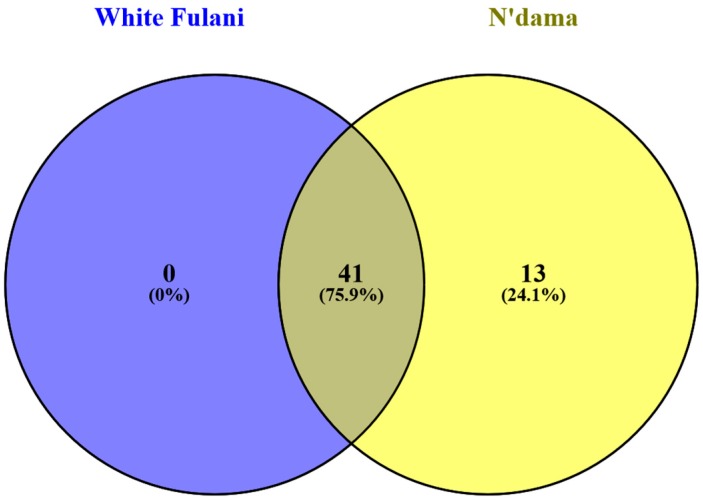
SNP overlap of the *CD14* gene promoter between White Fulani and N’Dama cattle. Forty -one SNPs were concomitant between White Fulani and N’Dama cattle while 13 were unique for N’Dama. No unique SNPs were identified for White Fulani.

N’Dama *CD14* promoter region was found on ARS-UCD1.2: 7:51765824-51766809 while that of White Fulani was located on ARS-UCD1.2 7:51765824-51766419. Both were found on chromosome 7 of the bovine genome ARS-UCD1.2 assembly of the Ensembl database. Our search for variations within the *CD14* gene promoter revealed a total of 54 and 41 variants at the *CD14* promoter region for N’Dama and White Fulani, compared to the reference genome, respectively (Table 1), with N’Dama presenting with significantly more variants. A total of 41 variants were common among both breeds, with N’Dama possessing additional 13 unique variants that were completely absent in White Fulani (Figure 2). These unique SNPs and their positions on the ARS-UCD1.2 genome assembly are shown in (Table 2). All the detected SNPs (common and unique) were found at the upstream region of the *CD14* gene. Using sequence ontology (http://www.sequenceontology.org/) classification, upstream gene variants are those located at the 5’ of a gene, classified as intergenic (SO:0001631), and subsequently subdivided into 2 KB upstream variant (SO:0001636) and 5 KB upstream variant (SO:0001635). The variants found in this study are classified under the 2 KB upstream type, belonging to structural variants, which change one or more sequence features.

### 3.3. Prediction of SNP Effects, Nucleotide Diversity, and Transcription Factor Binding Sites

**Figure 3:**
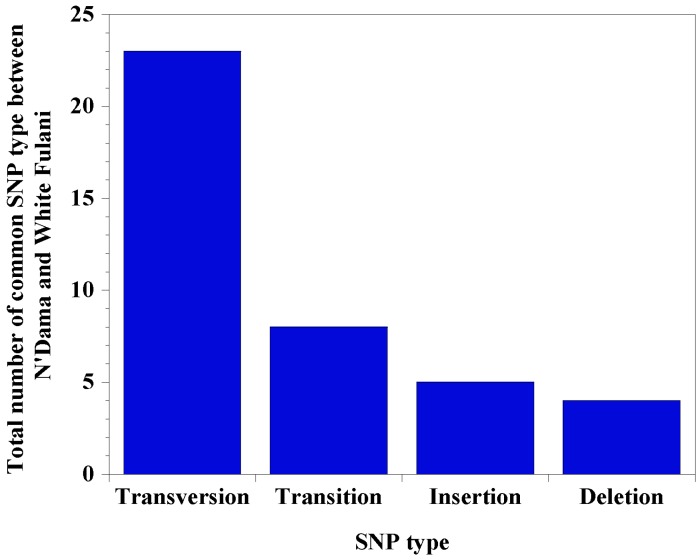
Total number of common SNP types between N’Dama and White Fulani. SNP types in the study include transversions (56.1%), followed by transitions (19.5%), insertions (12.2%), and deletions (9.8%). One SNP (2.4%) is a tandem repeat (not shown).

**Figure 4:**
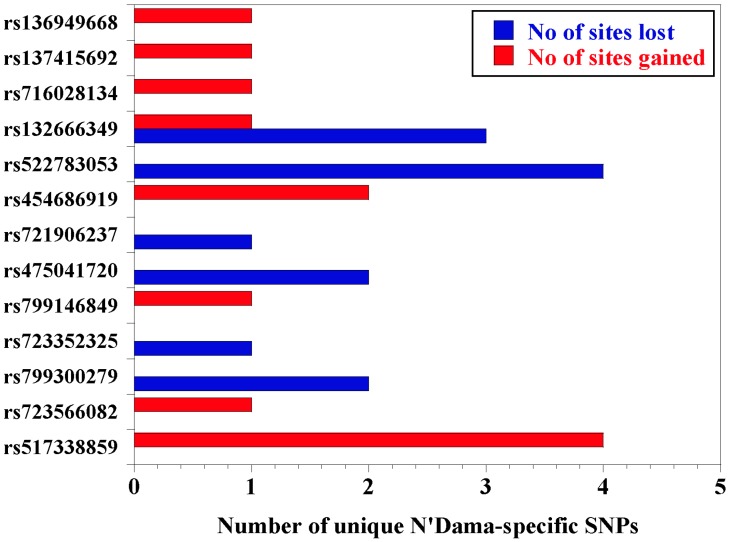
Effect of identified N’Dama-specific single nucleotide polymorphisms on transcription factor binding sites.

We determined the distribution of single nucleotide polymorphisms within the sequence and predicted the potential effects on transcription factor binding sites. Distribution of common variants reveal that there are 78% single nucleotide polymorphisms (*n =* 32), 10% deletions (*n =* 4), and 12% insertions (*n =* 5) in White Fulani, whereas N’Dama specific variants have 92% single nucleotide polymorphisms (*n =* 12), 8% deletions (*n =* 1), and no insertions. Among the common SNPs, nucleotide substitution showed that there were more transversions (70.9%) than transitions (29.3%) (Figure 3), with a similar trend observed for N’Dama specific SNPs. We report a total of 69 and 89 transcription factor binding sites lost and gained, respectively, due to the common SNPs at the *CD14* promoter region of both N’Dama and White Fulani (Table 1). However, N’Dama specific single nucleotide polymorphisms produced a total of 13 transcription factor binding sites lost and 12 new ones gained (Figure 4; Table 3). In all, N’Dama has a total of 101 new transcription factor binding sites and lost 82. Interestingly, we found the effect of some SNPs having overlapping transcription factor binding sites. A list of the common transcription factor binding sites in both breeds is presented (Supplementary Table S2), with significantly related N’Dama-specific binding sites listed in Table 3. Higher new transcription factor binding sites found in both breeds are attributed to high transverse mutations among the identified single nucleotide polymorphisms. By computational prediction, six of such polymorphisms with dbSNP numbers; *rs440282053*, *rs450747566*, *rs482000686*, *rs459318293*, *rs442402639,* and *rs456854916*, have a higher number of newly formed transcription factor binding sites ranging from 6 to 12.

### 3.4. miRNAs Identification and Functional Classification

To infer potential miRNAs and their functions at the promoter region of both breeds, we carried out prediction of miRNA binding sites within the core promoter region of the *CD14* gene. We found two common miRNA binding sites (bta-miR-2381 and bta-miR-2340) for the two animals, in addition to bta-miR-12032 which is specific for N’Dama, and bta-miR-22-5p and bta-miR-22-3p that are specific for White Fulani (Table 4). Moreover, we examined the presence of single nucleotide polymorphism within each mature miRNA sequences. We found that bta-miR-2381 is highly polymorphic having seven nucleotide variants. On the other hand, N’Dama-specific miRNA (bta-miR-12032) possessed only one variant, and none for White Fulani. Altogether, these variants are regarded as mature miRNA variants, or transcript variants, due to their location within the sequence of mature miRNA. Gene ontology shows that these miRNAs are implicated in various biological processes including the downregulation of gene expression through RNA-induced silencing complex.

### 3.5. Allelic, Genotypic, and Haplotype Estimates of Three Loci in N’Dama CD14 Promoter 

**Figure 5:**
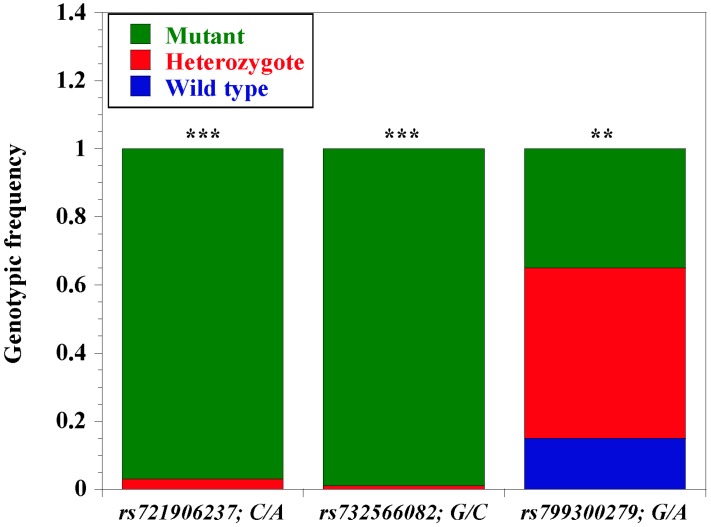
**Genotypic frequency of three selected N’Dama-specific *CD14* gene promoter single nucleotide polymorphism**. We elucidated the genetic diversity of three (out of 13) selected N’Dama-specific SNPs (*n =* 103) using Taqman SNP genotyping assay (ThermoFisher). Blue bars: Wild type variant; red bar: Heterozygotes; green bars: Mutant variants. The three SNPs are *rs721906237*; C/A; *rs732566082*, G/C; and *rs799300279*, G/A; *** significant (*p* < 0.0001); ** (*p* < 0.001); * (*p* < 0.01).

To understand the distribution of allelic frequencies, three loci were randomly chosen for subsequent TaqMan real-time PCR analysis of an unrelated N’Dama population. As shown in Table 5, a general observation from our analysis showed a predominance of the mutant variant from all the three loci. For the SNP locus *rs721906237*; C > A, mutant allele A has a higher frequency of 0.97 (195) compared to the wild type allele C with 0.03 (7). Likewise, locus *rs723566082*; G > C presents a higher frequency of the mutant variant C at 0.99 (205) as compared to the wild type allele G with 0.01 (1). It is also observed that locus *rs799300279*; G > A presents 0.6 (121) mutant allele A and 0.4 (81) wild type allele G. Furthermore, Table 5 showed a significant genotypic diversity among the three loci. We observed that there is complete lack of the wild genotype C/C (*rs721906237*; C > A) and G/G (*rs723566082*; G > C) from the two *CD14* promoter loci in all the N’Dama animal samples. The two loci present higher frequencies of homozygous mutant genotypes A/A and C/C with an estimate of 0.97 and 0.99, respectively (Figure 5). On the other hand, *rs799300279*; G > A locus has 0.35 homozygous mutant genotype A/A, 0.5 heterozygote genotypes A/G, and 0.15 wild type genotype G/G. Notably, heterozygote genotype A/G is significantly higher compared to the other types. To examine the relationship/co-occurrence of the three loci for evolutionary inference, haplotype estimates were constructed for the three single nucleotide polymorphism loci. Overall, five haplotype groups (H1 to H5) were constructed from the analysis. Haplotype combination H1 (*ACA*) and H2 (*ACG*) were significant within the population while H5 (*CCG*) is a rare combination (Table 6).

## 4. Discussion

Gene promoter regions have been tagged as significant players determining the steady-state accumulation of mRNA with differences attributed to SNPs and regulatory elements. *CD14* gene is a major part of the innate immune system, and one of essential receptors needed to initiate an adequate response to infection [13,14,15]. Encounter with antigens trigger innate immune responses mediated by host genetic variations alongside additional benefits arising from prior exposure to such stimuli that may direct tolerance or susceptibility [18,35]. In this study, we dissected the genetic variation within the core promoter region of bovine *CD14* gene to elucidate a deeper understanding of trypanosomosis tolerance in cattle. We present possible evidence of selection signature within the *CD14* gene promoter region in disease tolerant N’Dama that is not seen in susceptible White Fulani. 

We identified a total of 54 single nucleotide polymorphisms within the *CD14* gene promoter region of the animals, which were mapped to the recent *Bos taurus* genome in the Ensembl database (also available in the dbSNP database). The high SNP density in this region is not unusual, considering the plasticity of noncoding regions compared to coding regions. Reports have shown that human *CD14* gene promoter is significantly polymorphic, and this polymorphism has been shown to influence its biological activities, including disease susceptibility [36,37]. Likewise, there are a handful of reports linking *CD14* gene polymorphism with disease susceptibility in cattle [14,38,39,40]. To this end, we postulate that the extensive polymorphisms observed in this report will play significant roles in gene expression, regulation of bovine *CD14,* and possibly disease tolerance. In addition, we report 13 N’Dama-specific *CD14* gene promoter variants that were not found in trypanosusceptible White Fulani, revealing possible evidence of localized selective sweep between the animal breeds, and potentially related to disease tolerability. Notably, the fact that almost all N’Dama animals were genotyped with predominantly mutant alleles from the 2 loci (*rs721906237*; C/A and *rs723566082*; G/C) indicate a complete mutation at this region, possibly due to its earlier exposure to disease on introduction to sub-Saharan Africa, potentially traceable to development of tolerance. Although locus *rs799300279*; G > A showed an incomplete mutation as there were more heterozygote alleles, this possibly indicates that the locus is still under selection pressure. As previously argued [4,6,8], this is an indication of an evolutionary adaptation event in N’Dama that is not observed in White Fulani. 

SNP diversities are valuable tools for understanding mutational molecular mechanisms and identification of disease susceptibility [3]. Our report shows that the majority of identified SNP variations are transversions (70.9%) rather than transitions (29.3%) [41]. The variations at both coding and noncoding regions of *CD14* gene have been reported to affect its surface expression on neutrophils and monocytes [13,15]. Although both SNP types in our study are regarded as point mutations from the regulatory region, transverse mutations at the coding region are known to significantly alter gene or protein expression than transition mutations [13,36]. The high frequency of transversions observed possibly indicates these SNPs are likely to cause pronounced effect on regulatory elements at the promoter region, resulting in changes in *CD14* expression, supporting DNA variability in the promoter region and association with disease tolerance [36,37]. This form of variation is potentially of the radical rather than conservative type, and evidence of selection, whereby transversions are more detrimental than transitions and predictive of mutational fitness effects [41,42,43], justifying our hypothesis. Therefore, these results provide additional evidence that polymorphisms of the *CD14* gene promoter may be one of the adaptation mediators of immune response to trypanosomosis. 

Published reports have shown that sequence changes at gene promoter regions are critically associated with disease phenotype, due to molecular evolution [36,37,44]. Our current result is contrary to reports in humans [36,44], where more transitional mutations (71%; C > T and G > A) than transversions (27%) were found, possibly a result of evolutionary diversity between the two species (cattle and humans). We have recently shown an extensively diverse *CD14* interactome with other immune-related genes among mammalian species [45]. As shown from our result, there is significant SNP diversity at the promoter region of *CD14* gene, indicating selection sweep between the disease tolerant N’Dama and susceptible White Fulani cattle. Other reports have shown significant association of *CD14* gene polymorphism with varying disease phenotypes, including monocyte surface expression in Canadian Holstein and Jersey cows during bovine mastitis [13], and in Chinese Holstein cows during bovine tuberculosis [15,38,46,47].

It is well known that gene promoter regions contain several transcription factor binding sites (TFBS) [48,49,50], and we hypothesized that higher SNP density reported in this region might cause loss of TFBS or generation of new ones, with possible biological impact, hence the need to predict the effects of each SNP on the TFBSs. Our *in silico* analysis reveal 37 out of 54 SNPs had significant impact on the predicted transcription factor binding sites, with 12% specifically found in N’Dama, which is expected, considering more SNPs were found in this animal. This breed-specific regulatory difference may interfere with the transcriptional control of *CD14* gene during downstream regulation in the animals [13,21]. We also found that some SNPs generated more TFBS than others, and as such likely to affect transcriptional activities, producing significantly more biological variations. An excellent strategy to further explore this observation would be to perform in vitro studies that investigate individual SNP effect on TFBS and gene expression during infection. There is a possibility that some SNPs with overlapping TFBS in these animals may have similar effects, with no biological relevance. However, some of the TFBS have been found to influence the expression of the adjacent or surrounding genes in some other systems [49,50,51]. Additionally, it has been suggested that sequence diversity in promoter regions might be an evolutionary indication, representing signatures of selection due to selective pressure [52,53,54]. Altogether, the presence of mutant alleles and regulatory motifs in N’Dama but not White Fulani strongly indicate an evolutionary adaptation of *CD14* gene for disease tolerance, and may be the underlying basis for disease susceptibility in White Fulani. 

Ibeagha-Awemu et al. [13] reported that nucleotide changes at the noncoding untranslated regions (UTR) increases the surface expression of *CD14* on monocytes and neutrophils among Canadian cattle. As reported in our miRNA prediction data, the core promoter region of *CD14* in both breeds contain miRNA binding sites, which are known to regulate the mRNA expression of the target gene [55]. Although predicted SNPs are present in the common bta-miR-2381 and N’Dama-specific bta-miR-12032, their effect on gene expression/regulation is unclear and may require an in vitro validation. Notably, gene ontology showed that these miRNAs are involved in multiple biological processes, including RNA-induced silencing and downregulation of gene expression. Several studies have shown the importance of miRNAs, including altering the expression of two or more target genes, miRNAs could induce translational repression or switch to translational activation [25,48,49,50]. Notably, we have earlier shown that *CD14* gene co-regulates or co-expresses with a list of other innate and adaptive immune system genes in cattle [40,56]. For example, *CD14* together with *TNF, LY96, LBP, MYD88, TLR-2, IL-18, TLR-4, CXCL8, CCL2, IFNγ*, and *IL-6* have been reported to initiate cellular response to bacterial lipopolysaccharide (LPS), cytokine production, regulation of immune system process, and chemokine metabolic process, etc. [8,40]. It is imperative therefore to examine the important pathways involved in host immune response for disease tolerance in cattle, since receptor activation and cytokine production are important for protection against disease. 

Other evidence has shown that regions of mutation are extremely sensitive, with genes and gene products that are functionally related [36,53,57,58,59]. The significant interactions of *CD14* gene with other functionally related genes indicate its importance in mediating immune response during disease condition in cattle. The predominant mutant allele in N’Dama animal may be a key to proper expression of *CD14* gene which is lacking in White Fulani. Therefore, such *CD14* molecule will provide an optimal and effective immune response against infection. On the other hand, a wild type *CD14* gene, as seen in White Fulani, establishes the foundation for lack of adaptation and subsequent inadequate immune response, and susceptibility to disease. This is a significant evidence that the wild type *CD14* allele in N’Dama animal may have undergone a complete mutation upon exposure to diseases at the earlier stages of life, which is lacking in White Fulani. There is the possibility that this variation may be responsible for the *CD14* expression and the subsequence activation/driver of other innate immune response gene such as Toll-like receptors. We expect that the higher mutant allele frequenting for the three loci of *CD14* gene promoter as seen in N’Dama is associated with a locally adapted immune response, primarily upon exposure to pathogen. 

## 5. Conclusions

Taken together, we conclude that the genetic diversity observed in the *CD14* promoter are attributable to selective sweep due to natural selection between the two breeds in this study. A better understanding of the underlying mechanism of immune response, potentially driven by evolutionary adaptation to an endemic environment is needed. *CD14* gene promoter region is important in regulating gene expression, which in turn drives the expression of other associated genes. This observation could be useful for possible drug or vaccine design purposes, or serve as basis for marker-assisted selection, providing useful information for conservation studies and selective breeding of cattle with increased resistance to infectious diseases.

## Figures and Tables

**Table 1:** Common single nucleotide polymorphisms (SNPs) information at the *CD14* gene promoter region between N’Dama and White Fulani cattle.

S/N	SNP ID	Position	Mutation	Substitution Type	Consequence on Transcription Sites	
					Number of Sites Lost	Number of Sites Gained
**1**	**rs471069687**	**53449742**	**C → A**	Transversion	2	3
2	rs438450850	53449670	C → T	Transversion	1	0
3	rs472947228	53449666	C → A	Transversion	0	1
4	rs452987322	53449661	G → C	Transversion	3	0
5	rs517148865	53449741	G → C	Transversion	1	1
6	rs442454268	53449654	G → A	Transition	2	2
7	rs522987651	51766435	-/C	Insertion	0	1
8	rs520985067	51766431	T/-	Deletion	1	0
9	rs473919411	53449641	T → A	Transversion	2	4
10	rs455092482	53449635	A → T	Transversion	4	2
11	rs438139354	53449628	A → C	Transversion	4	2
12	rs469547425	53449627	G → C	Transversion	4	1
13	rs452652871	53449622	T → C	Transition	1	2
14	rs515988451	51766408	-/G	Insertion	-	-
15	rs523833822	51766400	T/-	Deletion	-	-
16	rs518926579	51766395	-/G	Insertion	-	-
17	rs526768201	51766392	T/-	Deletion	-	-
18	rs452331337	53449596	G → T	Transversion	1	0
19	rs443071218	51766373	(T)12/(T)13/(T)14	Tandem repeat	-	-
20	rs439484127	51766372	-/T	Insertion	-	-
21	rs730878557	51766372	T/-	Deletion	-	-
22	rs440282053	53449583	A → T	Transversion	1	7
23	rs797368076	51766324	-/T	Insertion	-	-
24	rs210109064	53449421	G → A	Transition	2	1
25	rs473185091	53449334	C → T	Transition	1	1
26	rs521360374	53449277	C → T	Transition	1	2
27	rs454858160	53449264	G → T	Transversion	0	1
28	rs432371570	53449221	C → A	Transversion	1	3
29	rs466880145	53449214	A → C	Transversion	3	2
30	rs446947915	53449212	T → C	Transition	3	3
31	rs481617328	53449165	T → G	Transversion	1	1
32	rs467889921	53449162	G → T	Transversion	1	4
33	rs450747566	53449152	G → T	Transversion	0	11
34	rs482000686	53449147	G → C	Transversion	5	9
35	rs458853723	53449144	T → G	Transversion	5	2
36	rs438670802	53449139	G → T	Transversion	5	1
37	rs479468105	53449129	A → T	Transversion	1	0
38	rs459318293	53449109	T → C	Transition	2	7
39	rs442402639	53449098	A → C	Transversion	1	6
40	rs473930339	53449097	C → A	Transversion	5	2
41	rs456854916	53449093	T → C	Transition	5	12
					**Total: 69**	**Total: 89**

**Table 2:** N’Dama-specific single nucleotide polymorphisms identified at the *CD14* gene promoter region.

S/No	SNP ID	Position	Mutation	Substitution Type	Consequences on Transcription Sites	
					Number of Sites Lost	Number of Sites Gained
1	rs517338859	53449994	G → A	Transition	0	4
2	rs723566082	51766726	G → C	Transversion	0	1
3	rs799300279	51766718	A → G	Transition	2	0
4	rs723352325	51766710	C → T	Transversion	1	0
5	rs799146849	51766708	A → G	Transition	0	1
6	rs475041720	51766680	G → C	Transversion	2	0
7	rs721906237	51766666	C → A	Transversion	1	0
8	rs454686919	51766632	C → A	Transversion	0	2
9	rs522783053	51766588	G → T	Transversion	4	0
10	rs132666349	53449790	C → T	Transition	3	1
11	rs716028134	53449782	T → G	Transversion	0	1
12	rs137415692	53449769	A → C	Transversion	0	1
13	rs136949668	51766556	A/-	Deletion	0	1
					**Total: 13**	**Total: 12**

**Table 3:** List of transcription factor binding sites associated with N’Dama-specific *CD14* gene promoter SNPs.

TFBS	TFBS Information	Position	Strand	Matrix Simulation	Sequence
IRF2.01	Interferon regulatory factor 2	122	(−)	0.945	caatgaatgaaagtGAAAagtgaaa
ZNF219.0	Kruppel-like zinc finger protein 219	213	(−)	0.920	caggcCCCCcttccctgggattc
GCM1.03	Glial cells missing homolog 1 (secondary DNA binding preference)	222	(−)	0.851	gtgacCCCCcataga
PLAGL1.02	Pleiomorphic adenoma gene-like 1 (secondary DNA binding preference)	225	(+)	0.816	atggGGGGtcacacagagtcaga
KLF12.01	Krueppel-like factor 12 (AP-2rep)	338	(+)	0.938	ggtcccaGTGGttaagact
HMBOX.01	Homeobox containing 1	340	(+)	0.834	tcccagtgGTTAagact
ZTRE.01	Zinc transcriptional regulatory element	358	(−)	0.963	gtgGGAGtgcagtctta
E2F.02	E2F, involved in cell cycle regulation, interacts with Rb p107 protein	420	(+)	0.849	cagcagggcCAAAaaaa
OVOL1.01	Zinc finger transcription factor OVO homolog-like 1	438	(+)	0.891	aaatctGTTActttc
CP2.02	LBP-1c (leader-binding protein-1c), LSF (late SV40 factor), CP2, SEF (SAA3 enhancer factor)	442	(+)	0.846	aACTGttactttcttaata
CEBP.01	CCAAT/Enhancer Binding Protein	453	(−)	0.941	tttattaaGAAAgta
HMX2.03	Hmx2/Nkx5-2 homeodomain transcription factor	466	(−)	0.822	tgttctTTAAatgtgttta
HNF4.02	Hepatic nuclear factor 4, DR2 sites	519	(−)	0.775	gaagttggtctAAAGaacagcttcc
STAT6.01	STAT6: signal transducer and activator of transcription 6	529	(−)	0.899	stagTTCCagagaagaagt
RREB1.01	Ras-responsive element binding protein 1	588	(+)	0.840	cCCCAaaatatccag
AIRE2.02	Autoimmune regulatory element binding factor	589	(−)	0.885	actggatattTTGGg
BTEB3.01	Basic transcription element (BTE) binding protein, BTEB3, FKLF-2	610	(+)	0.948	ggaaattcagGGAGttgaa
RU49.04	Zinc finger transcription factor RU49, zinc finger proliferation 1 - Zipro1	683	(+)	1.000	aAGTAcc
CEBPB.01	CCAAT/enhancer binding protein beta	873	(−)	0.966	gaaatttcGCAAtga

Abbreviations; TFBS: Transcription Factor Binding Sites. The red colors in the TFBS sequences are the conserved motifs in the binding sites that specifically binds to the transcription factors.

**Table 4:** List of micro RNAs (miRNAs) and associated SNP variants identified at the *CD14* gene promoter region.

	miRNA Variants	no of SNP	Position	Sequence	Accession Number	Variants IDs
bta-miR-2381	C/A C/G T/A/C T/G C/G T/G G/C	7	1-19	CAGGCUGCUCUGUGCUUGGCU	MIMAT0011929	*rs472162209* *rs442506482* *rs462738247* *rs476414754* *rs438710923* *rs458831864* *rs478292631*
bta-miR-2340	-	-	3-17	GGACUUCCCUGGUGGUCUUGUG	MIMAT0011875	N/A
bta-miR-12032	T/G	1	180-200	UCUGGCCUGGAGAAGCCCUGG	MIMAT0046725	*rs438484117*
bta-miR-22-5p	-	-	15-36	AGUUCUUCAGUGGCAAGCUUUA	MIMAT0003826	N/A
bta-miR-22-3p	-	-	53-73	AAGCUGCCAGUUGAAGAACUG	MIMAT0012536	N/A

Abbreviations; N/A: Not applicable. Red letters indicate SNP position in the miRNA sequence. miRNAs: micro ribonucleic acids; red color nucleotides depict the mutation spots within the miRNA sequences.

**Table 5:** Genetic diversity of selected N’Dama-specific polymorphisms in the *CD14* gene promoter region.

Polymorphism	Genotype	Genotypic Count (*n =* 103)	Frequency	Significance
***rs721906237***	C/C	0	0	NS
	C/A	7	0.03	*
	A/A	94	0.97	***
***rs732566082***	G/G	0	0	NS
	C/G	1	0.01	NS
	C/C	102	0.99	***
***rs799300279***	G/G	15	0.15	*
	G/A	51	0.50	***
	A/A	35	0.35	**
	**Allele**	**Allelic Count**	**Frequency**	**Significance**
***rs721906237***	C	7	0.03	***
	A	195	0.97	***
***rs732566082***	G	1	0.01	***
	C	205	0.99	***
***rs799300279***	G	81	0.40	***
	A	121	0.60	***

NS: Not significant; *** significant (*p* < 0.0001); ** (*p* < 0.001); * (*p* < 0.01).

**Table 6:** Estimated haplotype frequencies of selected N’Dama-specific *CD14* gene promoter SNPs.

Haplotype	Haplotype Definition			Haplotype Frequency	*p* Value
	*rs721906237* *(C/A)*	*rs723566082* *(G/C)*	*rs799300279* *(A/G)*		
H1	A	C	A	0.5594	0.002
H2	A	C	G	0.4012	0.046
H3	C	C	A	0.0346	0.990
H4	A	G	A	0.0049	0.068
H5	C	C	G	0	NS

Abbreviations: NS: Not significant. SNPs at the three loci *rs721906237*, *rs723566082,* and *rs799300279* define five different haplotypes. C/A, G/C, and A/G denote the alleles at the three loci.

## Supplementary Materials

The following are available online at https://www.mdpi.com/2073-4425/11/1/112/s1, Table S1: Primer sequences for the genomic amplification of CD14 gene promoter, Table S2: List of common transcription factor binding sites associated with *CD14* gene promoter region in both animals.
