# Supplementary material for: SNP Diversity in CD14 Gene Promoter Suggests Adaptation Footprints in Trypanosome Tolerant N’Dama (Bos taurus) but not in Susceptible White Fulani (Bos indicus) Cattle
Source: Genes (Basel). 2020 Jan 19;11(1):112. doi: 10.3390/genes11010112 (PMC7017169; doi:10.3390/genes11010112)
Supplement: Supplementary file 1 [file genes-11-00112-s001.pdf]

Supplementary Table 1: Primer sequences for the genomic amplification of *CD14* gene promoter.

S/N	Primer sets	Sequences (5'- 3')	Size (bp)
1	CD14 promoter	F: ACACACCTGGAGAAGGCAA<br>R: TCCAAGGGCTAGTTCCAG AG	553
2	CD14 promoter	F: CAATTCCTGGTCAGGGAACTAA<br>R: GGCAGCCTCTGAGAGTTTATGT	613

Supplementary Table 2: List of common transcription factor binding sites associated with *CD14* gene promoter region in both animals.

Matrix Family	Transcription factor information	Regulatory sequence	Position	# in WF	# in ND	*p*-value
XBBF	Regulatory factor X 5	ggGTTGccattgccttctc	7	1	1	0.010
TEAF	TEA/ATTS DNA binding domain factors	acccATTCcagta	22	1	1	0.145
NFAT	Nuclear factor of activated T-cells 5	gcctGGAAaatcccaagga	40	1	2	0.142
ESRR	Estrogen-related receptor alpha	ctggaaaatcccAAGGacagagg	42	1	1	0.108
IRFF	Interferon regulatory factor 7	tgaaaagtGAAAgtgaagtcgctca	113	2	3	0.141
PRDF	Positive regulatory domain I binding factor	gaaaagtGAAAgtgaagtc	118	2	1	0.102
HUB1	Zinc finger protein 282 (HTLV-I U5 repressive element-binding protein 1)	TTTCacttttcactt	126	2	2	0.148
HOXF	T-cell leukemia, homeobox 2	agaagggAATGgcaaccca	152	1	1	0.100
SMAD	Smad4 transcription factor involved in TGF-beta signaling	gtgGTCTtgcc	176	1	1	0.145
NFKB	Nuclear factor kappa B/c-rel	ctGGGAttctccagg	185	1	1	0.143
PAX6	PAX-4/PAX-6 paired domain binding sites	ctggagaatcCCAGggaag	186	1	2	0.144
ZTRE	ZTRE motifs (1 bp spacer), ZNF658 binding site	ccttccctGGGAttctc	189	2	3	0.153
GLIF	GLIS family zinc finger 2	caggCCCCccttccctg	197	1	1	0.141
SRFF	Serum response element binding factor	gacccCATAgacggcagcc	217	2	1	0.013
SREB	Sterol regulatory element binding protein 1 and 2	aagTCACctcagttg	250	1	1	0.092
NF1F	Myoblast determining factors	agtagcCAGCagcagcg	267	1	2	0.104
PAX3	PAX-3 binding sites	tgtgTCTCgctgctgctgg	272	1	2	0.086
CAAT	Cellular and viral CCAAT box	gtggCCAAtgaagat	311	1	1	0.105
CTCF	CTCF and BORIS gene family, transcriptional regulators with 11 highly conserved zinc finger domains	tccacmccccctgcagtGGGAgtgcagt	354	1	2	0.088
KLFS	Kidney-enriched kruppel-like factor, KLF15	cccactgcaGGGGtgtgga	362	1	2	0.024
BCL6	POZ/zinc finger protein, transcriptional repressor, translocations observed in diffuse large cell lymphoma	caaTTCCtggtcaggga	385	2	1	0.144

CSEN	Downstream regulatory element-antagonist modulator, Ca2+-binding protein of the neuronal calcium sensors family that binds DRE (downstream regulatory element) sites as a tetramer	tgGTCAgggaa	392	2	1	0.108
MEF3	MEF3 binding site, present in skeletal muscle-specific transcriptional enhancers	ggcTCAGggaact	394	1	2	0.148
E2FF	E2F, involved in cell cycle regulation, interacts with Rb p107 protein	cagcagggcCAAAaaaa	418	1	2	0.047
MEF2	Myocyte-specific enhancer factor 2	cagcagggcCAAAaaaa	420	1	2	0.064
HNF6	Onecut homeodomain factor HNF6	aaaaaaAATCtgttact	433	1	1	0.058
MYBL	Cellular and viral myb-like transcriptional regulators	ttaagaaagtAACAgattttt	436	1	1	0.144
DMRT	Doublesex and mab-3 related transcription factor 5	aaaatctGTTActttcttaat	437	1	1	0.112
CART	Reproductive homeobox 6, placenta specific homeobox 1	tttctTAATaaacacatttaa	449	1	2	0.088
FAST	FAST-1 SMAD interacting protein	aaatgtgttTATTaaga	451	1	1	0.143
FKHD	Fork head domain factors	tcttaatAAACacattt	451	1	1	0.147
NKXH	Homeo domain factor Nkx-2.5/Csx, tinman homolog low affinity sites	tttctTAATaaacacattt	458	1	1	0.145
HBOX	Homeobox transcription factors	acttgttcTTTAaatgtgt	460	1	1	0.072
AP1R	MAFB/Kreisler basic region/leucine zipper transcription factor (half site)	ttaaagaacaagTCAGcaaaaaca	490	1	1	0.133
BEDF	Zinc finger, BED-type containing 4; polyG binding sites	aggacgtGGGGggga	495	2	1	0.138
ZBED	Zinc finger, BED-type containing 4; polyG binding sites	aggacgtGGGGggga	495	2	3	0.139
EGRF	EGR/nerve growth factor induced protein C & related factors	ggacgTGGGggggaagctg	496	2	3	0.112
NR2F	Nuclear receptor subfamily 2 factors	gaagttggtctAAAGaacagcttcc	507	1	2	0.114
HEAT	Heat shock factor 1	actccaagggstagttCCAGagaag	531	1	1	0.121
HMTB	Human muscle-specific Mt binding site	aggtATTTa	597	1	1	0.128
NKRF	Nuclear factor-kappaB repressing factor	attTCCTcagg	603	1	1	0.108
BZIP	Hepatic leukemia factor	tgaacttggGTAAtagg	631	1	1	0.062
HOMF	Hematopoietically expressed homeobox, proline-rich homeodomain protein	taataggaagtTAATaaca	633	1	1	0.057
NKX6	NK6 homeobox transcription factors	gaagTTAAtaacaac	639	1	1	0.092
HNF1	Hepatic nuclear factor 1	aGTTAttttttttttg	642	2	1	0.104
MYT1	MYT1 C2HC zinc finger protein	aaaAAGTtatgga	667	1	1	0.029
OCT1	Octamer-binding factor 1	gtTATGgaaagtacc	672	1	1	0.095
GCMF	Chorion-specific transcription factors with a GCM DNA binding domain	ccctgCCCCtatccc	701	2	2	0.061

SP1F	Stimulating protein 1, ubiquitous zinc finger transcription factor	gatagGGGCagggggaca	703	1	1	0.129
AP2F	Transcription factor AP-2, alpha	caagCCTGggggcat	729	1	1	0.145
SNAP	snRNA-activating protein complex	aTCTCcttttctccaaccc	748	1	1	0.145
RORA	Orphan nuclear receptor rev-erb alpha (NR1D1), monomer binding site	cactgcctggGTCAgaggaatagag	799	1	1	0.141
RXRF	RXR heterodimer binding sites	gcctgGGTCagaggaatagaggcct	803	1	1	0.145
STAT1	Signal transducer and activator of transcription	ccctTTCCaggcaacatcc	849	1	2	
STAT3	Signal transducer and activator of transcription 3	tcaaTTCCtggtcagggaa	877	1	1	0.141
ETSF	Ets variant 1	aatttccaGGAAgagtagcga	880	1	2	0.136
MZF1	Myeloid zinc finger protein MZF1	gtGGGGagtga	925	1	2	0.083
PBXC	Pre-B-cell leukemia homeobox 3	gtggggagTGACagggt	925	2	2	0.039
HOXH	Meis1a and Hoxa9 form heterodimeric binding complexes on target DNA	TGACagggttcaaag	933	1	2	0.058
CHRF	Cell cycle gene homology region (CDE/CHR tandem elements regulate cell cycle dependent repression)	ttctTTGAaccct	937	1	2	0.106
STAT5	Signal transducer and activator of transcription 5	agggTTCAaagaaggggga	937	2	2	0.018
BARB	Barbiturate-inducible element	gttcAAAGaaggggg	940	1	1	0.146
PLAG	Pleomorphic adenoma gene 1	aaGGGGgagcaaagcgacttccc	948	1	1	0.059
RBPF	Mammalian transcriptional repressor RBP-Jkappa/CBF1	acccTGGGaagtc	963	1	1	0.068
PARF	Thyrotrophic embryonic factor / hepatic leukemia factor	cagggTTACataaactc	970	2	2	0.148

WF: White Fulani; ND: N’Dama.
